# MLH1 focus mapping in the guinea fowl (Numida meleagris) give insights into the crossover landscapes in birds

**DOI:** 10.1371/journal.pone.0240245

**Published:** 2020-10-05

**Authors:** Lucía del Priore, María Inés Pigozzi

**Affiliations:** INBIOMED (CONICET-UBA), Facultad de Medicina, Universidad de Buenos Aires, Buenos Aires, Argentina; University of California San Francisco, UNITED STATES

## Abstract

Crossover rates and localization are not homogeneous throughout the genomes. Along the chromosomes of almost all species, domains with high crossover rates alternate with domains where crossover rates are significantly lower than the genome-wide average. The distribution of crossovers along chromosomes constitutes the recombination landscape of a given species and can be analyzed at broadscale using immunostaining of the MLH1 protein, a component of mature recombination nodules found on synaptonemal complexes during pachytene. We scored the MLH1 foci in oocytes of the chicken and the guinea fowl and compared their frequencies in the largest bivalents. The average autosomal number of foci is 62 in the chicken and 44 in the guinea fowl. The lower number in the guinea fowl responds to the occurrence of fewer crossovers in the six largest bivalents, where most MLH1 foci occur within one-fifth of the chromosome length with high polarization towards opposite ends. The skewed distribution of foci in the guinea fowl contrast with the more uniform distribution of numerous foci in the chicken, especially in the four largest bivalents. The crossover distribution observed in the guinea fowl is unusual among Galloanserae and also differs from other, more distantly related birds. We discussed the current evidence showing that the shift towards crossover localization, as observed in the guinea fowl, was not a unique event but also occurred at different moments of bird evolution. A comparative analysis of genome-wide average recombination rates in birds shows variations within narrower limits compared to mammals and the absence of a phylogenetic trend.

## Introduction

Cytological crossovers (COs) are visualized as chiasmata which function as physical connections between homologous chromosomes. These physical ties counteract the spindle forces providing the tension necessary to ensure regular disjunction of homologs at meiosis I. In addition to this mechanistic role, COs build new heritable allelic variants increasing the genetic variation in the progeny [1]. The factors that regulate the frequency and spatial distribution of CO events are numerous and, in some cases, incompletely characterized. Among vertebrates, most efforts have concentrated on mammals to investigate how the number of COs and their distribution along chromosome arms vary within and between species [2, 3]. Mammalian karyotypes display great diversity in number and morphology [4] and these variations have a significant effect on the CO patterns [5, 6]. Extending the broad-scale analyses of crossing over to other vertebrate groups can help to determine if CO patterns are more stable in a context of limited genomic/chromosome rearrangements. An example of such a group is birds since they have less variation in the number of chromosomes than mammals and other vertebrate groups do. A typical avian karyotype has a 2n of 76–80, consisting of a few large-to-intermediate-sized chromosomes and many small (<10 Mb) chromosomes. Also, avian karyotypes are more stable because interchromosomal rearrangements are rare, except in certain groups (e.g., Psittaciformes and Falconiformes) where it is clear that karyotypes are highly rearranged [7–9]. Domestic Galloanserae (ducks, fowls and relatives) are one of the bird groups with lower rates of chromosomal rearrangements [10], with chicken chromosomes closely resembling the putative ancestral karyotype (PAK) of birds [11, 12]. For these reasons and the accessibility of meiocytes for experimental studies, they are good candidates to look at broad-scale CO patterns in a background of low karyotype/genomic variability.

**Fig 1:**
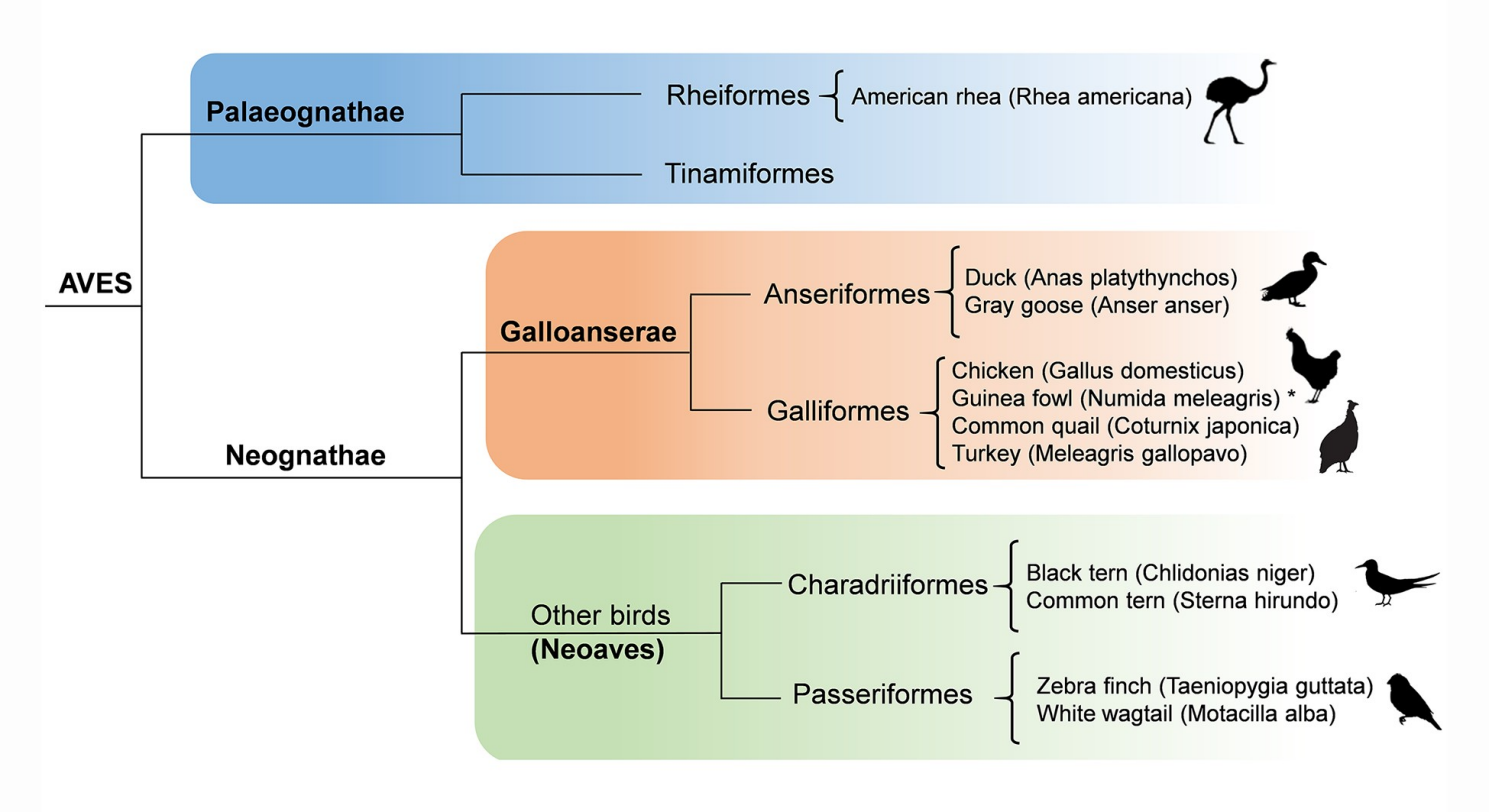
Simplified avian phylogenetic tree of avian groups with cytological crossover data. Except for Palaeognathae, the graph includes the avian orders with crossover data for at least two species. *Data for the guinea fowl are presented here. The tree is based on Prum et al., Nature 526(7574). doi: 10.1038/nature15697 (2017).

At the cytological level, CO events can be examined by observing chiasmata or through immunofluorescent detection of chromosome-associated protein complexes that are involved in recombination. Even though these methods are limited by microscopy resolution, they have the benefit to provide an overview of recombination across the whole genome, while at the same time being able to determine chromosome-specific patterns of crossing-over [13]. The immunocytological approach to analyze CO frequency and distribution along bivalents involves the detection of the protein MLH1, a component of late nodules associated with synaptonemal complexes (SCs) during pachytene [14, 15]. Current evidence in yeast and other organisms indicates that MLH1 foci tag most CO events, while a second type of COs (non-interfering) follows a molecular pathway lacking MLH1 [16, 17]. Because non-interfering events represent a small fraction of all recombination events, MLH1 focus maps in mammals provide good estimates of the frequency and distribution of COs along individual bivalents [18–23]. In birds, the presence of two types of CO pathways has not been investigated. However, the total number of MLH1 foci is very similar to the number of chiasmata in chicken oocytes and quail spermatocytes [24–26]. Since chiasmata are stable markers of COs, then MLH1 foci can be considered reliable cytological counterparts of COs in birds. Cytological COs in birds are limited to few species, but the analyzed taxa span the entire avian phylogeny, from a primitive ratite (Palaeognathae), to several species of the large group of Neoaves (Fig 1) [27–30].

Since the first report in the chicken [25], MLH1 foci studies in Galliformes were extended only to the common quail that, like the chicken, belongs to the family Phasianidae [24]. To further enhance the knowledge on the recombination landscape in Galliformes we turned to the guinea fowl as this species belongs to Numididae, a family that ranks among the oldest gallinaceous birds. Despite a divergence time of about 47 My, the chicken and guinea fowl macrochromosomes are largely syntenic, with few interchromosomal rearrangements in Numididae [31–33]. Studying the crossover pattern in the guinea fowl would help to understand what effect, in any, evolutionary distances have on recombination patterns in birds. To this end, we used immunolocalization of MLH1 in the oocytes of the guinea fowl and compare them with similar data in the chicken. We found that the MLH1 distribution in the largest SCs of the guinea fowl follows a distinctive pattern, with a disproportionate number of COs localized towards the chromosome ends and low frequencies in mid regions. This feature not only differs greatly from the pattern observed in the chicken but also departs from other Galloanserae. Here we analyze the variation of CO patterns among birds and discussed the differences observed with mammals.

## Materials and methods

### Birds

Fertile eggs from chicken (white layer breed, H&N International) and guinea fowl were purchased from commercial breeders and incubated under standard conditions until hatching. Handling and euthanasia of birds were performed according to protocols approved by the Animal Care and Use Committee of the University of Buenos Aires School of Medicine (EXP-UBA 0047533/16, Res 2116/16) following all institutional and national guidelines for the care and use of farm and laboratory animals.

### Synaptonemal complex spreads and immunostaining

SC spreads for measurements and MLH1 counts were obtained from six chicken females and two females of guinea fowl about 48 hours after hatching. The method to prepare SC spreads from avian oocytes has been previously described in detail [24, 34]. Briefly, the only functional ovary was minced in 100 mM sucrose at pH 8.5, and the released cells were suspended in more sucrose solution. About 30 μl of this cell suspension was dropped onto a layer of 1% paraformaldehyde fixative and 0.1% Triton X-100 on clean slides and left in a humid chamber for one hour. After fixation, slides were washed in Photoflo and air-dried. For immunofluorescence, the primary antibodies were anti-SMC3 (Chemicon, Millipore) at 1:1000 that labels the cohesin axes underlying the AE of the synaptonemal complexes and CREST human antiserum (Roquel Laboratories, Buenos Aires, Argentina) that binds to kinetochores at 1:100, and mouse anti-MLH1 (BD Pharmingen) at 1:100. The secondary antibodies were TRITC-labeled goat anti-rabbit, Cy3-labeled donkey anti-human, and FITC-labeled goat anti-mouse (Jackson ImmunoResearch) at 1:100. Immunostained spreads were scanned with 100X magnification objective at a fluorescence microscope suited with appropriate filter sets for each fluorochrome. Individual images for red and green fluorescence were acquired using an Olympus DP73 CCD camera. Images were corrected for brightness and contrast and merged using Adobe Photoshop 6.0.

### Image and data analysis

Measurements and MLH1 focus counts were done on composite images of immunostained SCs, centromeres, and MLH1 foci using the program Micromeasure [35], which records absolute and relative lengths and the positions of centromeres and MLH1 foci. MLH1 foci were counted in chicken oocytes in a previous study [25], but the antibodies available then were not suitable to label avian SC components, and presumptive SCs were visualized with DAPI staining after DNase I treatment. This precluded a precise analysis of MLH1 focus frequency along SC arms. Given the importance of the chicken as a reference species and to standardize this and future analyses, we now scored MLH1 foci in immunostained SC spreads from two-day-old females and then built individual MLH1-focus maps for the six largest autosomal bivalents. The construction of MLH1 maps was done following the procedures to build frequency histograms of foci and recombination nodules [19, 36, 37]. First, the average relative length of a given SC was determined and then multiplied by the average absolute length of a complete SC set in each species to obtain an average absolute length for each identified SC (S1 File). The relative position of each MLH1 focus was multiplied by the average absolute length for the appropriate SC to obtain the absolute (micrometer) position of each focus (S2 File). Data for each one of the six largest autosomal SCs were pooled and graphed in histogram form to demonstrate the pattern of MLH1 in each species. In the histograms, the interval size is 0.5 μm, fitting the resolution of the measuring device [38]. Average SC lengths were rounded to the most proximal decimal point. All MLH1 foci beyond the las interval on each arm were included in the last interval. Statistical comparison of MLH1 focus distribution was done using a Kolmogorov-Smirnov two-sample test. To do that we built cumulative frequency plots of MLH1 foci along the macrobivalents of each species. This type of representation offers a better comparison of frequency distributions and also allows for the statistical estimation of similarities [24, 39]. To produce cumulative distributions, the frequency of MLH1 foci was added along each SC starting from the tip of the short arm with distances expressed as a percentage of the SC length (S1 Fig). Statistical analyses were performed in Graph Pad Prism v. 6.01. Details on the statistics employed, degree of freedom and p-values are reported within Results or in the supplementary online files.

## Results

### SC karyotypes and MLH1 focus frequencies

**Fig 2:**
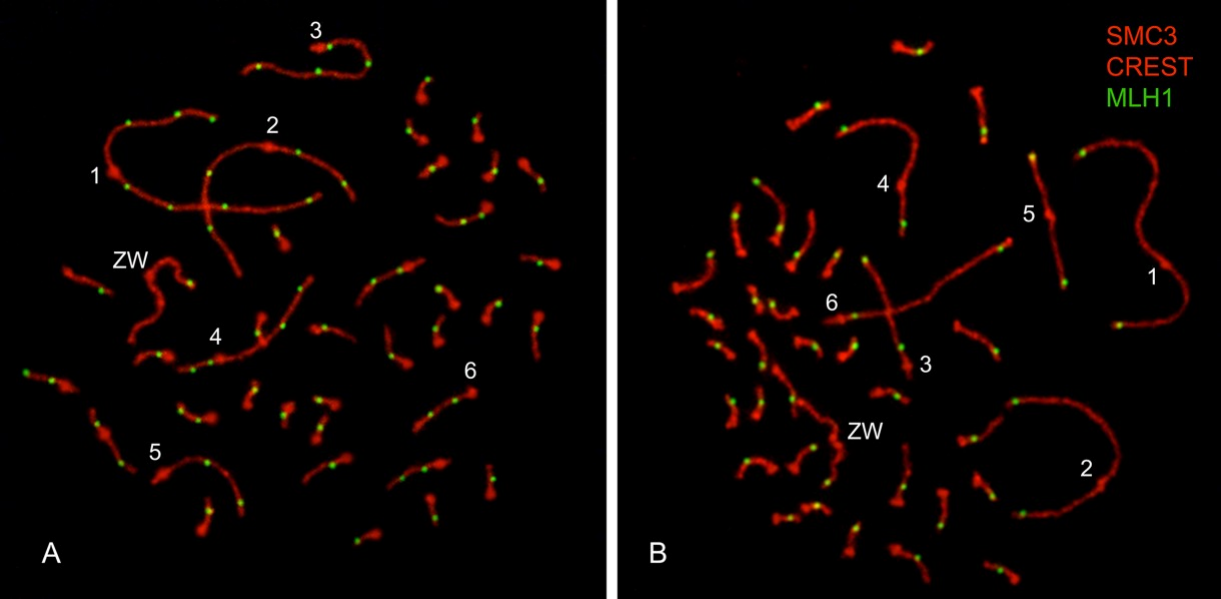
Immunolocalization of recombination events in chicken and guinea fowl oocytes. Chicken (A) and guinea fowl (B) immunostained oocytes showing the complete sets of synaptonemal complexes. The number of bivalents, including the sex pair, is 39 in the chicken and 38 in the guinea fowl. The six largest synaptonemal complexes are labeled with a number next to the centromere signal (red bulging dots). In the guinea fowl, foci on macro-SCs are predominantly located towards the chromosome ends.

Synaptonemal complex lengths and MLH1 focus numbers were scored in 138 chicken oocytes and 133 oocytes from the guinea fowl showing the full sets of SCs. Examples of the immunostained nuclei used for the analysis of MLH1 foci are shown in Fig 2. The pachytene sets in the chicken and the guinea fowl showed 39 and 38 bivalents, respectively, including the ZW pair. In both species, the six largest autosomal SCs can be identified based on their relative lengths and the positions of the centromeric signals (S1 File). These bivalents will be referred to here as macro-SCs or macrobivalents; the rest of the SCs decrease gradually in length and cannot be assigned with certainty to specific chromosome pairs in the mitotic karyotypes. The sex bivalent can be recognized because it has axial elements of different lengths or adopts a wavy appearance after a process of synaptic adjustment during pachytene [40].

The absolute lengths of the SC sets differ significantly between species, but the fractions represented by the six largest bivalents are very similar (Table 1). This result is in line with the homology between the macrochromosomes of both species (see below) and illustrates the linear relationship between the SC length and DNA content that is commonly observed in birds [25, 41].

**Table 1:** Average synaptonemal complex lengths and MLH1 foci in the chicken and the guinea fowl.

**Chicken**					
	**SC**	**Rel length (%)**	**Abs length (μm)**	**CI**	**N° foci**
**Macro-SCs**	1	15.2	28.5 ± 5.1	0.41	7.2 ± 1.6
	2	11.9	22.6 ± 5.5	0.35	5.9 ± 1.2
	3	8.7	16.3 ± 2.9	0.03	4.1 ± 1.0
	4	7.2	13.6 ± 2.6	0.24	3.7 ± 0.9
	5	5.5	10.4 ± 2.0	0.09	2.9 ± 0.8
	6	3.3	6.2 ± 1.4	0.05	1.7 ± 0.5
	1–6	51.8	99.1		25.5
**Micro-SCs**	7–13	2.6	5.6 ± 1.1		1.7 ± 0.6
	14–22	1.6	2.9 ± 0.6		1.1 ± 0.2
	23–38	1.0	1.9 ± 0.3		1.0 ± 0.1
**Autosomal set**			^a^ 188.5 ± 25.0		^b^ 62.1 ± 5.4
**Guinea fowl**					
	**SC**	**Rel length (%)**	**Abs length (μm)**	**CI**	**N° foci**
**Macro-SCs**	1	13.9	24.6 ± 2.2	0.39	2.3 ± 0.6
	2	11.2	19.8 ± 1.7	0.35	2.2 ± 0.5
	3	7.9	14.0 ± 1.0	0.03	2.0 ± 0.4
	4	7.6	13.4 ± 1.0	0.28	2.0 ± 0.3
	5	5.6	9.9 ± 0.8	0.48	1.9 ± 0.3
	6	5.1	8.9 ± 0.7	0.16	1.5 ± 0.3
	1–6	51.2	90.6		11.9
**Micro-SCs**	7–13	2.4	4.1 ± 0.6		1.1 ± 0.3
	14–22	1.7	3.0 ± 0.4		1.0 ± 0.1
	23–37	1.2	1.1 ± 0.3		1.0 ± 0.1
**Autosomal set**			^a^ 177 ± 10.7		^b^ 44.4 ± 1.6

Number of cells analyzed: 138 (chicken) and 133 (guinea fowl).

^a^ Means differ significantly. P value: < 0.0001; two tailed. t = 4.891 df = 269.

^b^ Means differ significantly. P value: < 0.0001; two tailed. t = 37.49 df = 269.

**Fig 3:**
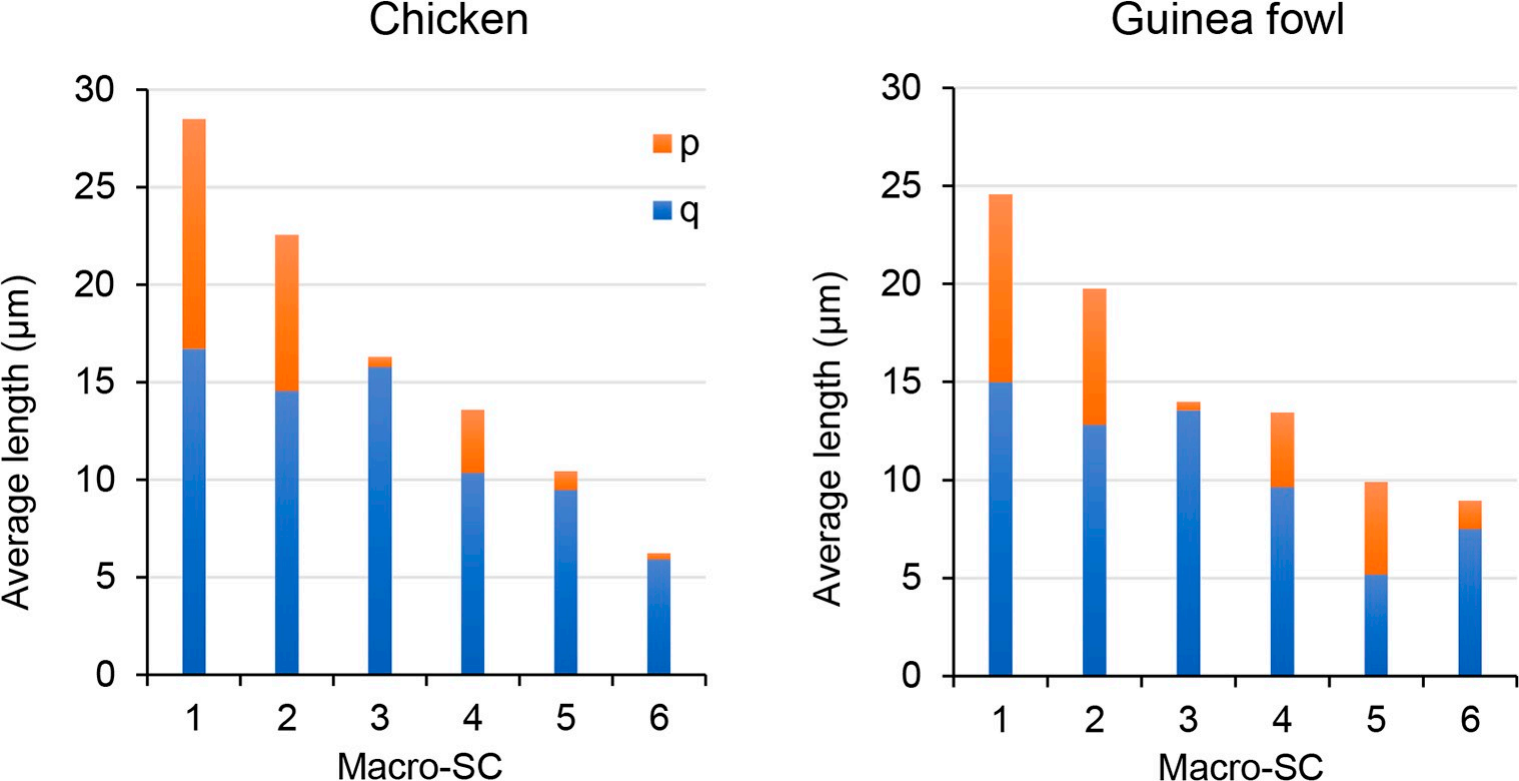
Graphic representation of the macro-SCs. Each bar represents a synaptonemal complex. The total height is the average SC length in micrometers. The short (p) and long (q) arms are shown in different colors to ease the comparison of the arm ratios.

In the chicken, the analysis of spermatocyte spreads by electron microscopy established the agreement of the macro-SCs with the mitotic chromosome pairs [42] and it is confirmed here using immunostained oocytes (Fig 2; Table 1). In the guinea fowl, we identified four submetacentric (SC1, SC2, SC4 and, SC5), and two acrocentric macro-SCs (SC3 and SC6), that correspond to the morphology of the macrochromosomes of the species in mitotic metaphases [43]. Cytogenetic and genomic studies showed that the ten largest chromosomes in the chicken and guinea fowl are highly conserved. Macrochromosomes 1 to 4 are homoeologous in both species and the same holds for chromosomes #6 of the guinea fowl and #5 of the chicken. The guinea fowl chromosome #5 is the product of a fusion, with the short arm corresponding to chromosome #6 and the long arm to chromosome #7 of the chicken. [31, 32]. These features are reflected in the macro-SC karyotypes where the four largest SCs share similar relative length and arm ratios, and the SC6 of the guinea fowl is comparatively larger than the chicken SC6 but similar in length to SC5 (Fig 3; Table 1).

Regarding the number of MLH1 foci per cell, we found mean values of 62 ± 5.4 and 44 ± 1.6 in the autosomal bivalents of the chicken and the guinea fowl, respectively (Table 1). Even though the distributions partially overlap, the means are significantly different at the statistical level. The genetic map length of the chicken calculated from the average MLH1 foci is therefore 3150 cM (62 autosomal foci + 1 focus in the ZW pair, multiplied by 50), and it is 2250 cM in the guinea fowl. The genome size (nuclear DNA content) in both species is close to 1.2 pg [44, 45], which implies about 1200 megabase pairs [46]. Thus, the genome-wide average recombination rates are 2.6 and 1.9 cM/Mb in the chicken and the guinea fowl, respectively.

**Fig 4:**
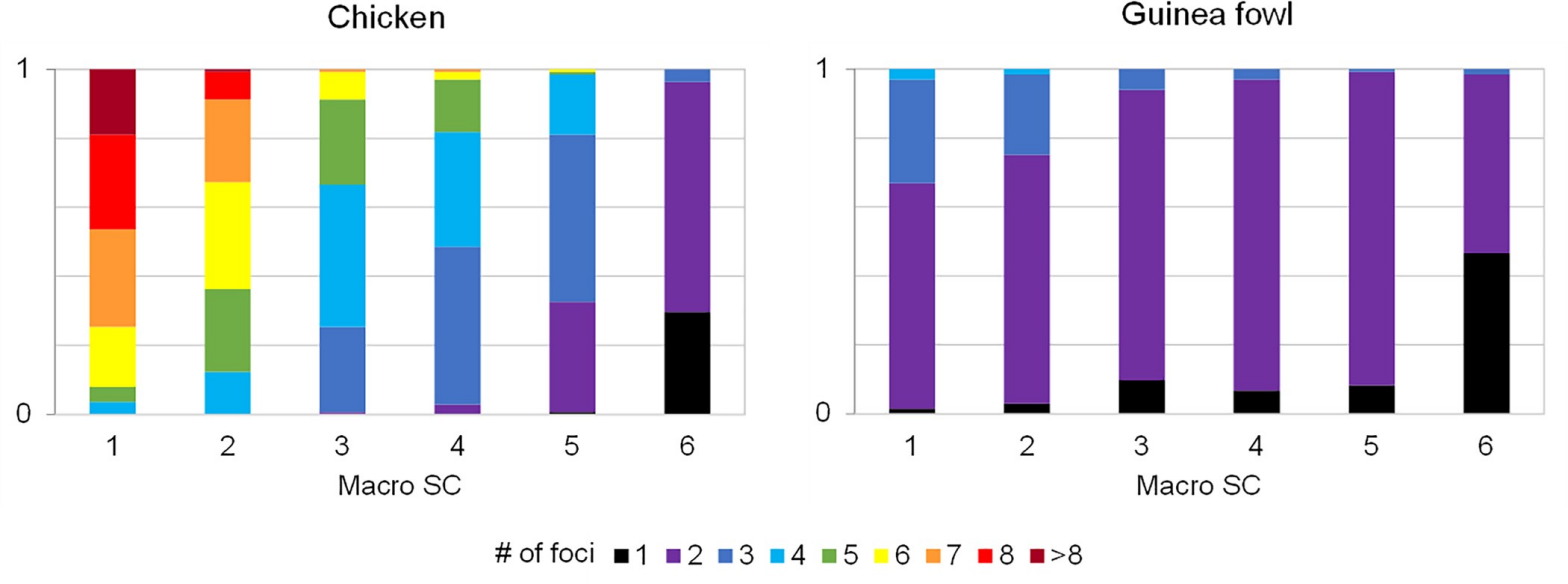
Comparative distribution of MLH1 foci in the six largest macrobivalents. Each column represents the distribution of SCs with n foci as a fraction of the cells analyzed in each species. Colors indicate bivalents with 1 to 8 or >8 foci. The color code is shown below the graph.

The interspecies difference of MLH1 foci is primarily explained by the number of foci in the macro-SCs 1 to 4 (Fig 4).

In the chicken, the two largest macro-SCs have 8 or more MLH1 foci in nearly half of the sampled nuclei, but in the guinea fowl the presence of more than two foci in these macrobivalents is unusual (Fig 4). The four largest SCs showed at least three foci in the chicken, while in the guinea fowl the most frequent observation was two foci per bivalent. The SC6 of the guinea fowl shows one or two foci in most cases, but its counterpart in the chicken (SC5) frequently have 3 o 4 foci. Macro-SCs without foci were not observed, and only a low number of microbivalents (< 0.1%) lack a focus in the sampled nuclei.

Intermediate-sized SCs, ranking 7 to 13 in measurements, also contribute to the different crossover levels since they have two foci in most chicken oocytes and only one in the guinea fowl (Table 1). In summary, both species have typical avian karyotypes with chromosomes that decrease gradually in size, and this is reflected in their SC lengths. At the same time, in both species, the longer SCs have more foci, but the upper limit of crossovers seems higher in the chicken than in the guinea fowl macro-SCs.

### The distribution of MLH1 foci in the macrobivalents

**Fig 5:**
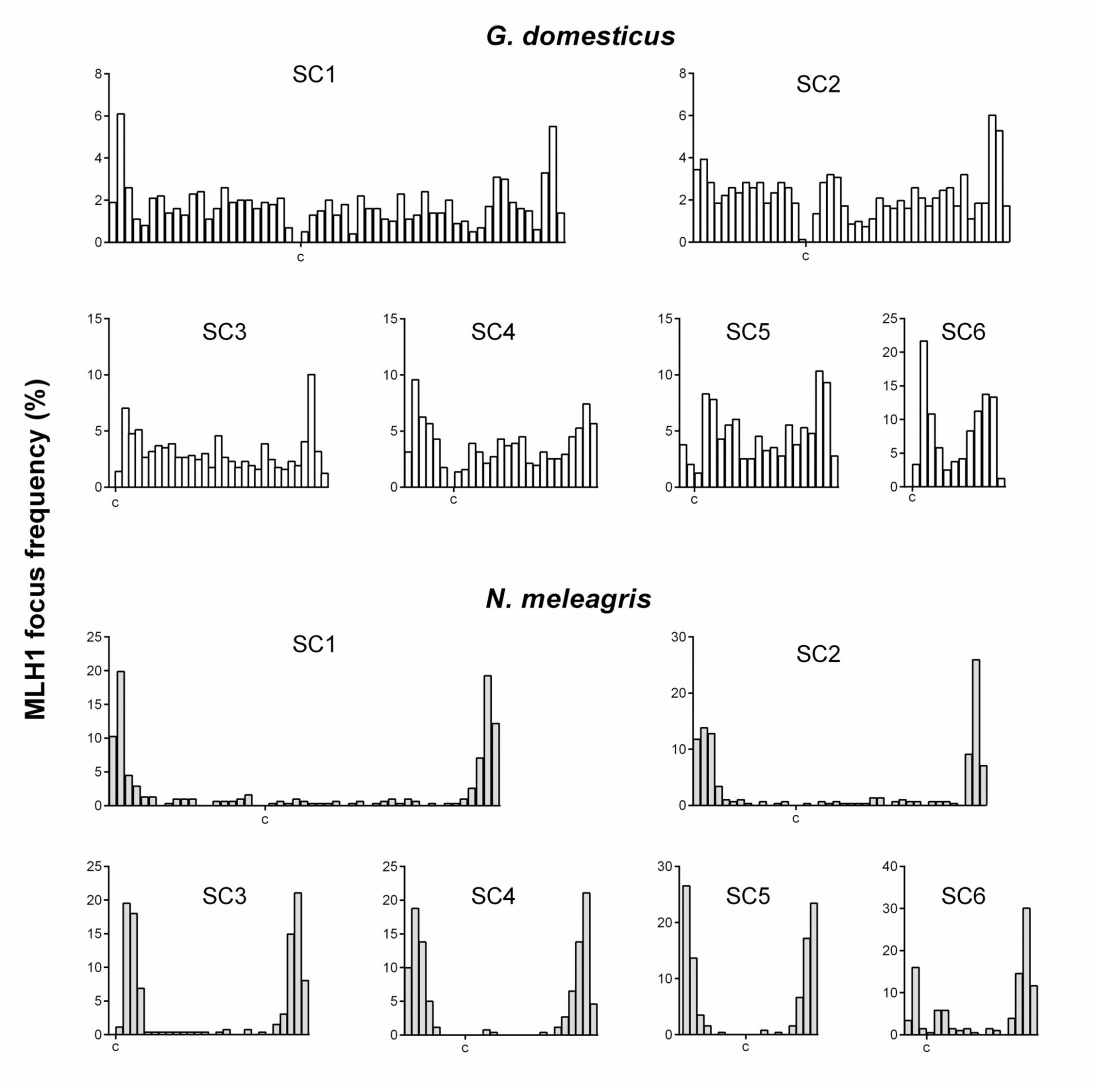
Distribution of MLH1 foci along individual macrobivalents of the chicken and the guinea fowl. For each bivalent, the *x*-axis indicates the positions of the MLH1 foci in micrometers measured from the centromere (c). The bin width in each histogram is equivalent to 0.5 μm. The *y-*axis indicates the proportion of MLH1 focus number in each interval.

In the macro-SCs of the chicken, MLH1 foci distribute rather evenly, with alternating peaks and valleys of recombination in the longest SC arms. In meta/submetacentric macrobivalents higher frequencies are observed near the telomeres; while, in the acrocentric elements, the intervals with higher CO frequencies are located on opposite ends of the long arm (Fig 5).

In the guinea fowl, MLH1 foci are accumulated towards the ends of macro-SC, with very low frequencies in the mid regions. Thus, both species share the feature that crossovers are more frequent towards the telomeres in the metacentric chromosomes and at opposite ends of the long arm of the acrocentric chromosomes. However, this trend is more noticeable in the guinea fowl where 86% of the COs (975 of 1131 foci) mapped within 20% of the chromosome length, including the proximal region of the long arm of the acrocentric SC3 (Fig 5; S2 File). The difference of focus distribution between the two species was verified by comparing the cumulative distribution of foci on homoeologous chromosomes using a Kolmogorov-Smirnov test (S1 Fig; Kolmogorov-Smirnov two-sample test, P < 0.0001 for all chromosomes).

## Discussion

### The chromosome number of numida meleagris is 2n = 76

In the recent genome assembly publication of the guinea fowl, the diploid number of the species was considered as not established [31], but close to 2n = 78 as reported from mitotic metaphases [32]. In our SC spreads we observed consistently 37 autosomal pairs plus the ZW bivalent, and therefore established a diploid number of 76. Variable counts of microchromosomes can occur even in high-quality mitotic chromosome spreads of birds. A source of this variation is the presence of a large secondary constriction in the NOR-bearing microchromosomes. As reported previously in the American rhea, this constriction makes a single element appear as two, dot-like microchromosomes in mitotic metaphases which causes an excess of one pair in the total chromosome count [47]. This is the most likely explanation for the different diploid numbers scored in our meiotic spreads vs. previous reports from mitotic chromosomes. The spreading method employed here is very reliable to count bivalents represented by their SCs, and it is especially useful in species with large numbers of small chromosomes. Pachytene nuclei rarely break down because the chromatin disperses gently in the fixative/detergent mix, keeping together all the bivalents within the same set. In the guinea fowl, no oocytes were observed with SC numbers over 38. Moreover, the same number of bivalents was counted, but not reported, in electron microscopy images of guinea fowl pachytene nuclei in a previous study of our laboratory [48]. The present results reinforce the value of SC analyses in birds to obtain precise chromosome counts.

### Localized and non-localized crossover distributions coexist in birds

**Fig 6:**
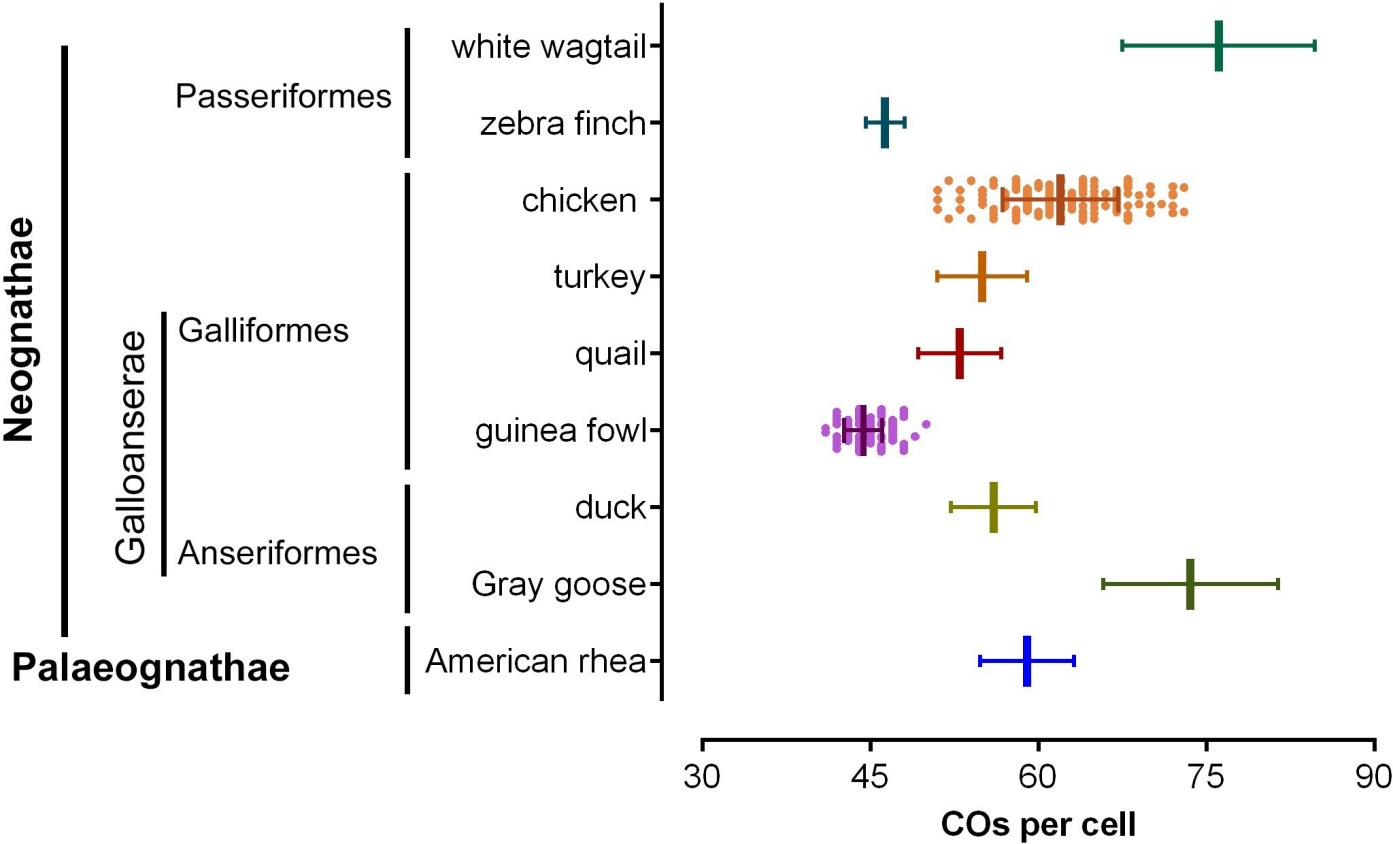
Number of COs per cell in birds showing the heterogenous variations between orders. Bars represent the average number of COs (vertical) and the standard deviation (horizontal) in each species. COs data are from MLH1 focus counts in all species except in the turkey that are from chiasmata in lampbrush chromosomes. Differences in CO numbers can be larger in species within the same order than across orders. Data source: chicken and guinea fowl, present study. American rhea, [27]; duck, [41]; Gray goose, [49]; turkey, [26]; common quail,[24]; zebra finch, [51]; white wagtail, [28].

It is known that the numbers of CO vary between species and even between sexes within the same species, so the difference reported here between the chicken and the guinea fowl should not be surprising. However, in three other domestic fowls -the chicken, the Japanese quail, and the turkey- the four largest macrobivalents have three or more COs with a relatively even distribution along SC arms [26, 41]. Moreover, the pattern of several COs along macrobivalents is also present in two Anseriformes, the duck and the Gray goose [41, 49]. Thus, the presence of multiple CO events along macrobivalents seems common within domestic Galloanserae. This pattern is not limited to this avian groups, as the presence of multiple, CO events more or less evenly spaced is also documented in birds from four other avian orders: the American rhea (Palaeognathae), pigeons (Columbiformes), two species of terns (Charadriiformes) and the white wagtail (Passeriformes) [27–29, 37]. Based on the present evidence this crossover pattern can be considered widespread among birds, and an ancestral feature maintained over 100 My, since these species represent the three main avian lineages: Palaeognathae, Galloanserae, and Neoaves (Fig 1) [50]. In this scenario of a long-time conserved CO landscape along macrochromosomes, the paucity of COs in the macro-SCs and their skewed distribution in the guinea fowl is, therefore, unexpected. Low numbers of MLH1 foci with strict localization were reported in the zebra finch macro-SCs, an estrildid bird of the order Passeriformes [51], and it was further evident following linkage map analysis in this species [52]. Localized recombination was also described in another estrildid bird, the long-tailed finch (*Poephila acuticauda*), based on analysis of genome-wide linkage disequilibrium data [53] and in the common swift (Apodiformes), by MLH1 focus mapping [54]. The CO pattern in Passeriformes is not conserved, since as previously mentioned the MLH1 focus map in the white wagtail showed the presence of numerous COs along macrobivalents resulting in the highest recombination rate known in birds [28]. The current knowledge CO distribution in birds, indicate that the broadscale recombination pattern in the macro-SCs shifted from multiple to localized CO events at different moments of avian evolution. These changes in CO frequency and distribution occurred in avian groups with highly variable biological adaptations, but also in others with more homogeneous traits, so the evolutionary events behind these changes are difficult to identify. This is also true for the molecular mechanisms behind the preferential localization of CO events observed in certain birds. The localized COs distribution observed in the guinea fowl and some passerines cannot be explained by the presence of heterochromatin. As in most birds, the macrochromosomes of the guinea fowl and the zebra finch do not have large blocks of heterochromatin and it is only present at centromeres [55–57]. The localized pattern of COs cannot be explained either by the so-called centromere effect [reviewed in 58], because the paucity of COs extends several micrometers beyond the centromere in metacentric chromosomes. Also, acrocentric macrobivalents have similar COs frequencies at centromeric and telomeric regions of the long arm (Fig 6). The organization of the DNA loops along meiotic axes is known to influence the number of recombination interactions that are solved as CO events [59]. In fact, birds have higher ratio of SC length per DNA amount than mammals and reptiles, and also have higher recombination rates, supporting the argument that DNA organization along the SC influences CO rates [60]. In birds like the guinea fowl or the zebra finch, with lower CO rates, the SC set lengths are shorter than in birds with higher CO rates (present results; [51]). These observations support the view that CO frequencies are directly related to the genome organization along the meiotic axes, as shown in mammals and other organisms, [3, 61–63].

### Genome-wide average recombination rates in birds show differences with mammals

The variations in the number of CO along the macro-SCs as described in the chicken and the guinea fowl, are an important influential factor in the global recombination frequencies in birds. This is so because chromosome numbers have small interspecies variations and because midi- and micro-SCs have one CO in most cases. Fig 6 shows the variation of CO numbers in birds of different phylogenetic groups that share the karyotypic and genomic features typical to most birds: high chromosome numbers, between 76 and 82 and small variation of the genome sizes that range between 1200 and 1400 Mbp [64]. The homogenous genome sizes in these species implies that the average number of CO represents also the genome-wide average recombination rates measured in cM/Mb.

It is observed that the differences in recombination rates can be larger within orders—the guinea fowl vs. the chicken (Galliformes) and zebra finch vs. the white wagtail (Passeriformes), than between orders (guinea fowl vs. zebra finch). Similarly, the American rhea and ducks show similar numbers of COs, while the number in the Gray goose largely exceeds the number in ducks.

At present, cytological CO maps are available for 13 species from 7 orders, with most data corresponding to domestic Galloanserae and Passeriformes. In this sample recombination rates vary between 1.8 to 2.6 cM/Mb [29, 30], and, as discussed above, they do not seems to be influenced by the phylogenetic positions. These features contrast with observations in mammals where the average recombination rates span an order of magnitude and are strongly influenced by the phylogenetic position, with lower rates observed in basal taxa compared to species of more recent divergence [2, 6, 65]. Genome-wide analysis of crossing over in more birds, especially from Neoaves are needed to speculate on the reasons why recombination rates are not lineage-dependent as in mammals.

## Supporting information

S1 File Data for Table 1 and Fig 3. Synaptonemal complex lengths (in micrometers); centromeric indexes and MLH1 foci in the chicken and the guinea fowl.
(XLSX)

S2 File Datapoints for Fig 5. MLH1 focus positions along synaptonemal complexes. Each value is the distance of one focus to the centromere expressed in micrometers.
(XLSX)

S1 Fig Cumulative frequency plots of foci in the chicken and the guinea fowl. The cumulative frequencies of foci on each synaptonemal complex (SC) are presented as a function of the distance to the telomeric end of the short arm (p) or to the centromere (cen). The distance is expressed as a fraction of the SC length on which the focus was located. For each bivalent, the P value represents the probability that MLH1 focus positions in the chicken and the guinea fowl stem from the same distribution (Kolmogorov-Smirnov two-sample test). The plots compare the homoeologous chromosomes or chromosome arms between species. The short arm of chromosome # 5 of the guinea fowl is homeolog to chromosome #6 of the chicken.
(TIF)
