# Supplementary figures and images for: MLH1 focus mapping in the guinea fowl (Numida meleagris) give insights into the crossover landscapes in birds
Source: PLoS One. 2020 Oct 5;15(10):e0240245. doi: 10.1371/journal.pone.0240245 (PMC7535058; doi:10.1371/journal.pone.0240245)

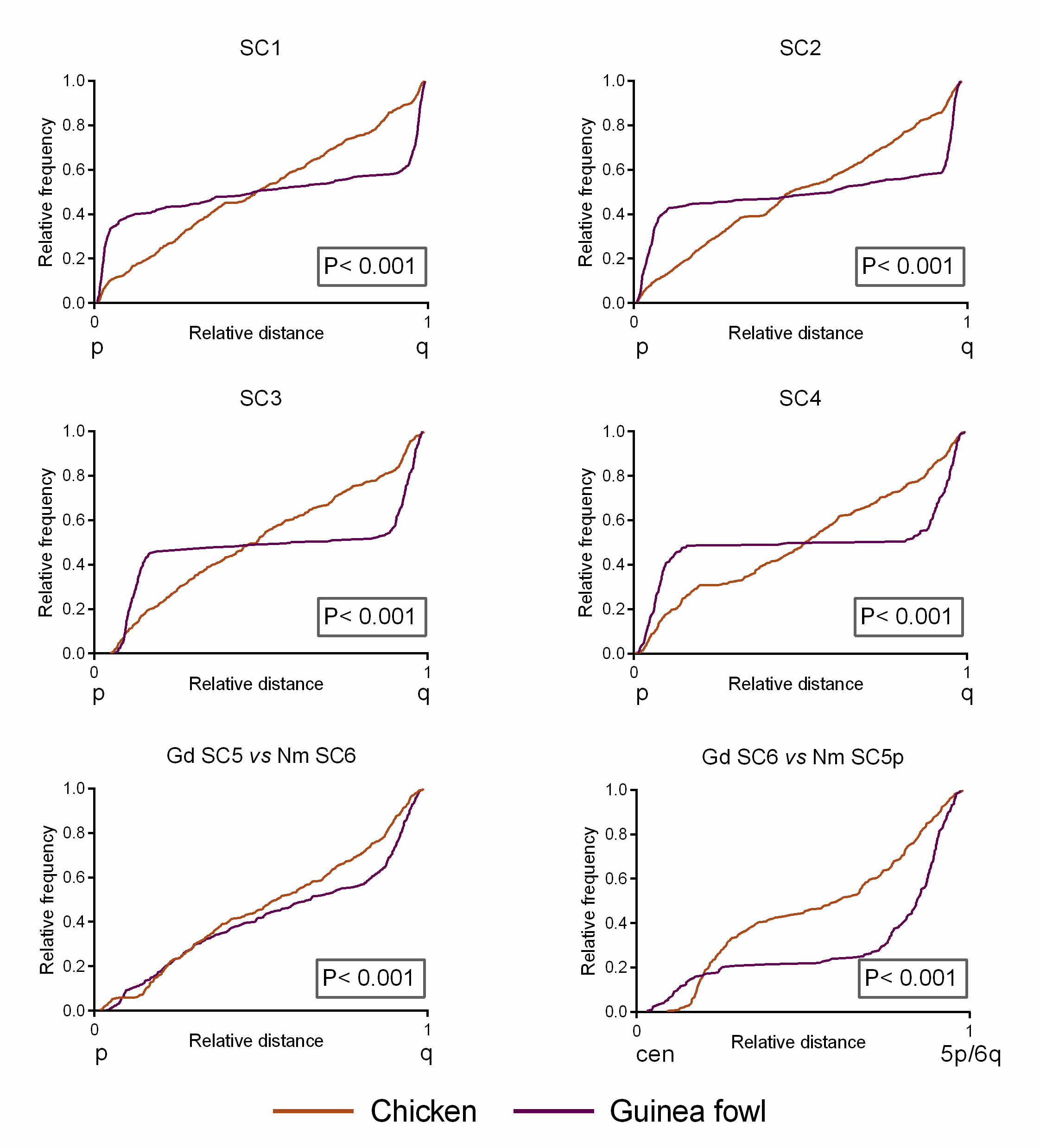

Supplement: S1 Fig — The cumulative frequencies of foci on each synaptonemal complex (SC) are presented as a function of the distance to the telomeric end of the short arm (p) or to the centromere (cen). The distance is expressed as a fraction of the SC length on which the focus was located. For each bivalent, the P value represents the probability that MLH1 focus positions in the chicken and the guinea fowl stem from the same distribution (Kolmogorov-Smirnov two-sample test). The plots compare the homoeologous chromosomes or chromosome arms between species. The short arm of chromosome # 5 of the guinea fowl is homeolog to chromosome #6 of the chicken. (TIF) [file pone.0240245.s003.tif]
